# The economic burden of influenza-associated outpatient visits and hospitalizations in China: a retrospective survey

**DOI:** 10.1186/s40249-015-0077-6

**Published:** 2015-10-06

**Authors:** Juan Yang, Mark Jit, Kathy S. Leung, Ya-ming Zheng, Lu-zhao Feng, Li-ping Wang, Eric H. Y. Lau, Joseph T. Wu, Hong-jie Yu

**Affiliations:** Key Laboratory of Surveillance and Early-warning on Infectious Disease, Division of Infectious Disease, Chinese Center for Disease Control and Prevention, 155 Changbai Road, Changping District, Beijing, 102206 China; Modelling and Economics Unit of Public Health in England, London, UK; Department of Infectious Disease Epidemiology, London School of Hygiene and Tropical Medicine, London, UK; WHO Collaborating Centre for Infectious Disease Epidemiology and Control, School of Public Health, Li Ka Shing Faculty of Medicine, The University of Hong Kong, 2/F North Wing, Patrick Manson Building, 7 Sassoon Road, Pok Fu Lam, Hong Kong Special Administrative Region China

**Keywords:** Influenza, Cost analysis, China

## Abstract

**Background:**

The seasonal influenza vaccine coverage rate in China is only 1.9 %. There is no information available on the economic burden of influenza-associated outpatient visits and hospitalizations at the national level, even though this kind of information is important for informing national-level immunization policy decision-making.

**Methods:**

A retrospective telephone survey was conducted in 2013/14 to estimate the direct and indirect costs of seasonal influenza-associated outpatient visits and hospitalizations from a societal perspective. Study participants were laboratory-confirmed cases registered in the National Influenza-like Illness Surveillance Network and Severe Acute Respiratory Infections Sentinel Surveillance Network in China in 2013. Patient-reported costs from the survey were validated by a review of hospital accounts for a small sample of the inpatients.

**Results:**

The study enrolled 529 outpatients (median age: eight years; interquartile range [IQR]: five to 20 years) and 254 inpatients (median age: four years; IQR: two to seven years). Among the outpatients, 22.1 % (117/529) had underlying diseases and among the inpatients, 52.8 % (134/254) had underlying diseases. The average total costs related to influenza-associated outpatient visits and inpatient visits were US$ 155 (standard deviation, SD US$ 122) and US$ 1,511 (SD US$ 1,465), respectively. Direct medical costs accounted for 45 and 69 % of the total costs related to influenza-associated outpatient and inpatient visits, respectively. For influenza outpatients, the mean cost per episode in children aged below five years (US$ 196) was higher than that in other age groups (US$ 129–153). For influenza inpatients, the mean cost per episode in adults aged over 60 years (US$ 2,735) was much higher than that in those aged below 60 years (US$ 1,417–1,621). Patients with underlying medical conditions had higher costs per episode than patients without underlying medical conditions (outpatients: US$ 186 vs. US$ 146; inpatients: US$ 1,800 vs. US$ 1,189). In the baseline analysis, inpatients reported costs were 18 % higher than those found in the accounts review (*n* = 38).

**Conclusion:**

The economic burden of influenza-associated outpatient and inpatient visits in China is substantial, particularly for young children, the elderly, and patients with underlying medical conditions. More widespread influenza vaccination would likely alleviate the economic burden of patients. The actual impact and cost-effectiveness analysis of the influenza immunization program in China merits further investigation.

**Electronic supplementary material:**

The online version of this article (doi:10.1186/s40249-015-0077-6) contains supplementary material, which is available to authorized users.

## Background

Influenza is associated with a substantial disease burden in China. Records show that between 2010 and 2011, 115 people per 100,000 population with influenza-related severe acute respiratory infections (SARI) were hospitalized, with this number increasing to 142 per 100,000 between 2011 and 2012 [1]. The influenza-related excess mortality rate for respiratory and circulatory diseases was estimated at 12.4 and 8.8 per 100,000 in China’s northern and southern cities, respectively [2].

The World Health Organization (WHO)’s Strategic Advisory Group of Experts on Immunization recommends that annual seasonal influenza vaccination be administered to pregnant women, children aged six to 59 months, the elderly, and those with underlying medical conditions [3]. However, China does not currently have a national government-funded seasonal influenza immunization program and the cost of vaccination is completely borne by the vaccinees. Uptake is extremely low; only 1.9 % of the population was vaccinated during the 2008–2009 influenza season [4]. In order for the influenza vaccination to be added to the national immunization schedule and be centrally funded, the National Health and Family Planning Commission and the Ministry of Finance need to consider the direct and indirect economic costs of influenza at the national level.

Several studies have explored the economic burden associated with seasonal influenza in China [5–9]. However, all but one [9] looked only at the direct medical costs and ignored the indirect costs to households and society due to productivity loss (even though these should be included according to the China Guidelines for Pharmacoeconomic Evaluations) [10]. Furthermore, these studies were all conducted in the tertiary hospitals of a few highly developed cities, such as Shanghai and Zhuhai, where costs associated with influenza are likely to be higher than those in the lower level hospitals in the more underdeveloped areas of the country [5–9].

To better quantify the economic burden of influenza patients across mainland China, we conducted a retrospective telephone survey to systematically assess the direct and indirect costs of influenza-associated outpatient visits and hospitalizations from a societal perspective.

## Methods

### Study design and enrollment of patients

The National Influenza-like Illness (ILI) Surveillance Network (ILINet) was established to monitor the activity of, as well as antigenic and genetic changes in, seasonal influenza viruses in China. This network includes 408 provincial- and prefecture-level Centers for Disease Control and Prevention (CDCs) and 554 sentinel hospitals situated in 31 provinces. All sentinel hospitals report the weekly number of ILI cases who seek medical care in their outpatient departments using the WHO standard ILI case definition (body temperature ≥38 °C with either cough or sore throat) in the absence of an alternative diagnosis. In each sentinel hospital, respiratory specimens are collected daily from the first one or two reported ILI cases and sent to local CDCs for influenza virus testing using virus isolation and/or reverse transcription polymerase chain reaction (RT-PCR) [2]. See Additional file 1 for information on the representativeness of this surveillance network.

The SARI Sentinel Surveillance Network (SARINet) was launched in 2009 to monitor severe diseases caused by influenza. It includes 10 tertiary hospitals located in 10 provinces and municipalities (Beijing, Heilongjiang, Zhejiang, Fujian, Shandong, Hunan, Guangdong, Sichuan, Yunnan, and Gansu). Respiratory specimens were collected from all inpatients with SARI for influenza virus testing using virus isolation and/or RT-PCR [7, 11].

We conducted a retrospective telephone survey between December 25, 2013 and January 11, 2014. Study participants were patients registered in the National ILINet and SARINet. The aim of the survey was to estimate the influenza-related direct medical costs (e.g. medication, examinations), direct non-medical costs (e.g. transportation, accommodation of patients and caregivers), and indirect costs (patients’ and caregivers’ loss of earnings). All laboratory-confirmed influenza cases or their caregivers who had telephone numbers registered in either the National ILINet or SARINet in 2013 were invited to participate.

### Telephone survey and data collection

The telephone surveys were conducted by Ipsos, a market research company in China. Well-trained interviewers from Ipsos used a computer-assisted telephone interviewing system (CATI), which involves using computerized questionnaires to do the interviews over the telephone and recording the answers directly into a computer, to conduct the surveys. A research assistant from the China CDC supervised the interviews. All phone calls were conducted at an Ipsos call center.

Family caregivers provided information on behalf of patients aged below 16 years. For other patients, data were collected from either the patients themselves or from their family caregivers. After explaining the objectives of the survey, we verified the demographic (e.g. name, age, gender, address) and clinical (e.g. date of onset, hospital visit date) details of each patient. Once this information was verified and the interviewee was able to recall a corresponding influenza episode, information about the costs related to that episode were elucidated. The questionnaire (see Additional file 2) included questions on: 1) general health conditions (e.g. having specific chronic diseases, duration of the corresponding influenza episode); 2) costs associated with outpatient treatment and hospitalization (e.g., number of outpatient treatments and hospitalizations, length of stay, costs of prescribed medications and examinations, transportation and accommodation, number of family caregivers and days of caring); and 3) cost of self-medication. We asked respondents to provide total medical costs based on the amounts specified on their medical bills (which included both out-of-pocket expenses for medical care as well as payments by insurers). If respondents could not precisely recall information such as the duration of hospitalization or cost, they were asked to provide a range. Each telephone number was dialed up to four times on different days before being classified as unreachable.

### Data analysis

Data analyses were conducted using R version 3.0.3 (R Project for Statistical Computing, Vienna, Austria). We calculated the direct medical, direct non-medical, and indirect costs stratified by age (<5, 5–14, 15–59, and ≥60 years); gender; presence of underlying medical conditions associated with increased risk of hospitalization or death if infected by influenza as listed in the WHO guidelines (these include chronic obstructive pulmonary disease, asthma, diabetes, chronic cardiac disease, chronic renal disease, chronic liver disease, chronic neurological disease, chronic hematological disorder, and tuberculosis, etc.) [12]; urban or rural residence (see Additional file 3 for what classifies an urban or rural area) [13], level of hospital (see Additional file 4 for definition of hospital levels); geographic region (Northeast, North, Northwest, East, Central, Southwest, and South); and virus type (influenza A, influenza B, and influenza untyped).

Transportation costs for those who used private vehicles were estimated by multiplying fuel cost per kilometer (0.650 RMB [14, 15] and 0.055 RMB [16] for petrol-powered cars and electric vehicles, respectively) by distance.

Productivity losses of patients and their caregivers were calculated using the human capital method. The average daily income per capita in 2013 as the value of the lost workdays was used. The average daily income per capita was assumed to be equal to the annual per capita income in 2013 (stratified by province, and by rural and urban area) (see Additional file 5) [17] divided by 365 days. For patients aged below 16 years, the annual per capita income was assumed to be zero. The cost of school days lost due to illness was not included. If family caregivers indicated that they took leave from work to care for patients, this was also considered. All costs were expressed in US dollars using the exchange rate in 2013 (1 US$ = 6.1956 CNY) [18].

Despite the skew in sample distributions of costs, the costs were summarized using the arithmetic mean and standard deviation (SD) so they could be used in economic evaluations [19]. We used the Wilcoxon rank-sum test or the Kruskal-Wallis test for group comparisons. We presented medians and interquartile ranges (IQR) of costs in Additional file 6. For qualitative variables, the chi-square test was used. We used bootstrap (1,000 replications) multiple linear regression to analyze the determinants for total costs. To partially correct for potential non-response bias, we presented the costs weighted by age group, region, and level of hospital using the population structure of the National ILINet and SARINet as a reference.

On the basis of this cost estimate and the influenza-associated excess hospitalization rate from hospital-based SARI surveillance in Jingzhou city, Hubei province [1], we estimated the population-level economic burden of influenza-associated hospitalizations in Jingzhou in 2013.

When we compared our estimates with those in previous studies, we inflated all costs in US dollars in previous studies for the given year to US dollars in 2013 using the country-specific consumer price index [20].

### Sensitivity analysis

As mentioned above, if respondents could not precisely recall certain details, they were asked to provide ranges (e.g. costs of prescribed medications and examinations). The lower limits of these ranges were used in the baseline analysis to ascertain the economic burden of influenza-associated outpatient and inpatient visits. We also conducted a sensitivity analysis to test the impact of uncertainty on the economic burden of patients by using the upper limits of the ranges.

### Cost validation

In the present study, we used patient-reported medical costs because of the difficulty of accessing accounts in all sentinel hospitals. Although patients receive medical bills that document the total costs including both out-of-pocket expenses for medical care and costs paid by insurers, recall bias may have still occurred. To validate patient-reported costs, we conducted a review of hospital accounts in three selected hospitals (Second Hospital of Fuzhou, Fujian Maternal and Child Health Care Hospital, and Hunan Provincial People’s Hospital), which accounted for 5 % of inpatients in our survey. Hospital accounts were reviewed in March–April 2015 for registered episodes of these patients.

### Ethical considerations

Ethical approval (no. 201417) was obtained from the Institutional Review Board of the China CDC before the survey began. Verbal informed consent was obtained from all respondents. For subjects younger than 17 years of age, parents/legal guardians were requested to give verbal informed consent.

## Results

### Telephone survey

**Fig. 1:**
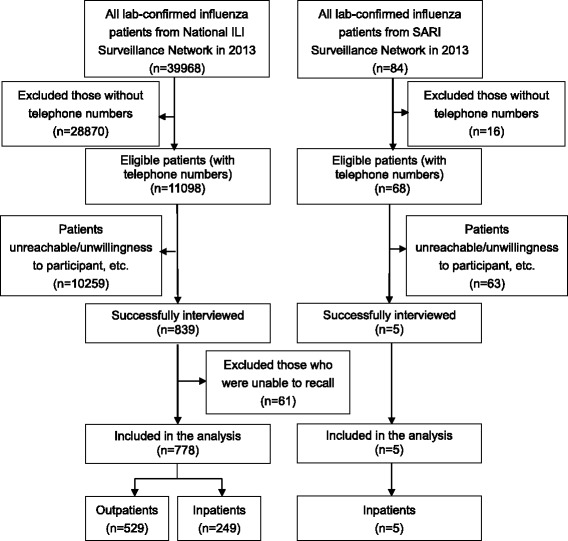
Flow chart showing the number of influenza patients included in the study

**Fig. 2:**
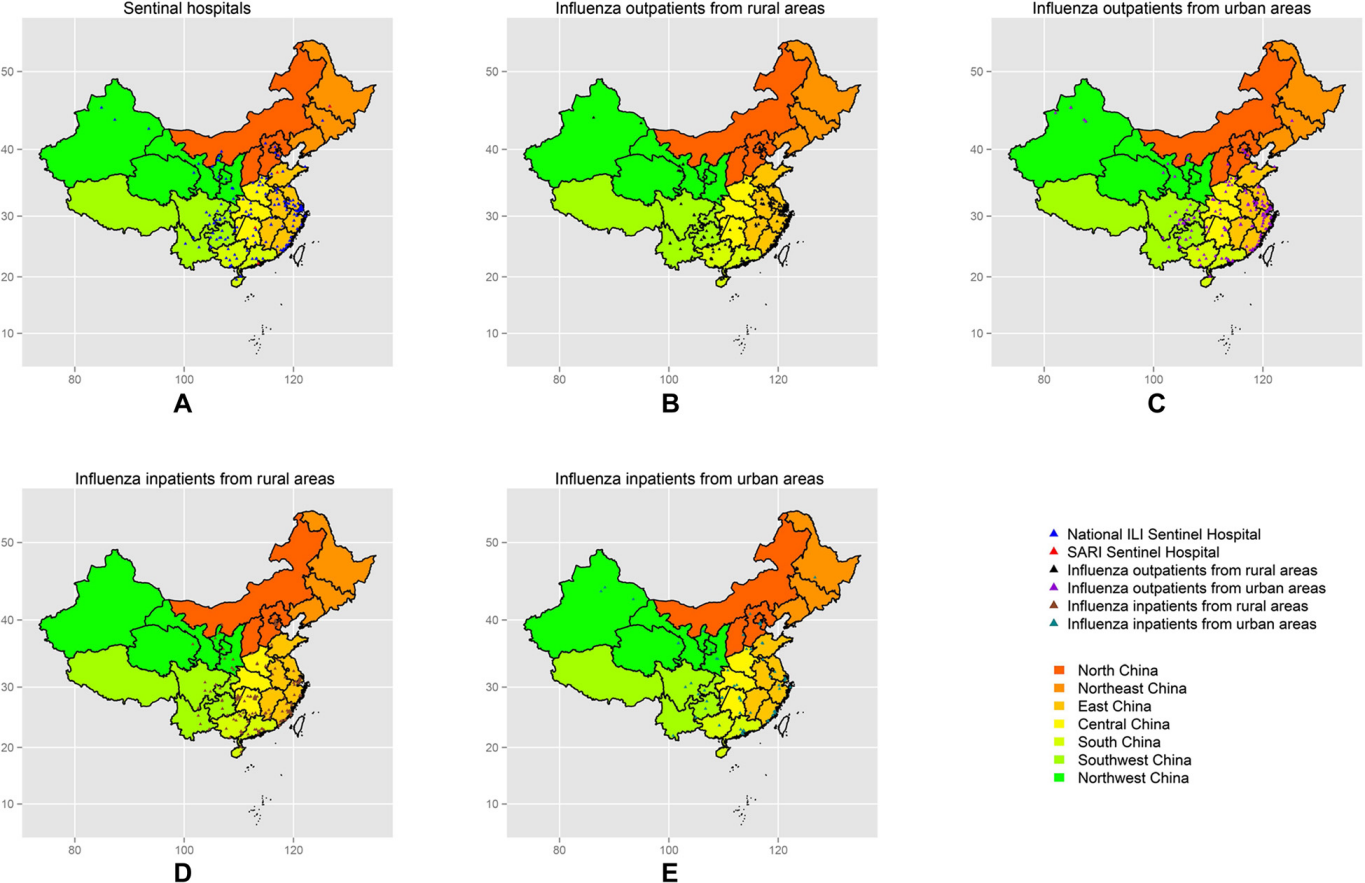
Geographic distribution of participating patients and the sentinel hospitals where they sought treatment. **a**: Geographic distribution of the sentinel hospitals where the participating patients were registered. **b**: Geographic distribution of participating influenza outpatients from rural areas. **c**: Geographic distribution of participating influenza outpatients from urban areas. **d**: Geographic distribution of participating influenza inpatients from rural areas.E: Geographic distribution of participating influenza inpatients from urban areas

In 2013, a total of 39,968 and 84 laboratory-confirmed influenza patients were registered in the National ILINet and SARINet, respectively. Of these, 11,098 and 68 patients from the National ILINet and SARINet, respectively, had their telephone numbers registered and were eligible to participate in this survey. We invited all these patients to participate and successfully interviewed 7.6 % (839/11,098) and 7.4 % (5/68) patients from the National ILINet and SARINet, respectively. Reasons for non-participation included language barriers, unwillingness to be interviewed, and being unreachable by telephone. Among the respondents recruited from the National ILINet, 61 were unable to recall a relevant influenza episode and were thus excluded from the study. In summary, the subjects for our study comprised five hospitalized cases from the SARINet, and 529 outpatients and 254 inpatients (inpatients who were hospitalized within two weeks of outpatient treatment) from the National ILINet (see Fig. 1). Figure 2 shows the geographic distribution of our subjects and the sentinel hospitals where they sought treatment. The median time between disease onset and interviews was 46 days (IQR: 20–103 days).

### Demographic characteristics of enrolled patients

Table 1 shows the demographic characteristics of the enrolled patients. There were statistically significant differences in age, region, and hospital level but not in gender between laboratory-confirmed influenza patients registered in the surveillance networks who participated in the study and those who didn’t (see Table 1). From the 783 included patients, 73.7 % were <15 years old, 54.7 % were male, 60.7 % were from tertiary hospitals, and 77.8 % were from urban areas. Almost half of the patients (47.8 %) resided in East China. The median ages of participating influenza outpatients and inpatients were eight years (IQR: five to 20 years) and four years (IQR: two to seven years), respectively. A larger proportion of inpatients (52.8 % [134/254]) had underlying diseases than outpatients (22.1 % [117/529]). The mean number of outpatient department visits was 2.1 (SD 1.7). Among the influenza inpatients, 12.6 % (*n* = 32) were hospitalized more than once; the average number of hospitalizations was 1.2 (SD 0.6) and the average duration of hospitalization was 9.0 (SD 4.9) days. The average lengths of stay were 7.7 (SD 3.6) days, 8.2 (SD 3.7) days, and 7.9 (SD 3.3) days for the first, second, and third hospitalization, respectively.

**Table 1:** Characteristics of included patients and other lab-confirmed influenza patients from surveillance networks in the survey

Characteristic	Included patients in the survey	Other lab-confirmed influenza patients from surveillance networks	Test for difference in groups	*p*-value
	(*n* = 783)	(*n* = 39269)		
Median age, years (inter-quartile range)	7 (3–15)	9 (4–27)	Rank-sum test	<0.001
Age group, years (%)			Chi-square test	<0.001
< 5	266 (34.0)	11854 (30.2)		
5–14	311 (39.7)	12113 (30.8)		
15–59	187 (23.9)	13653 (34.8)		
≥ 60	19 (2.4)	1649 (4.2)		
Male (%)	428 (54.7)	21568 (54.9)	Chi-square test	0.913
Region (%)			Chi-square test	<0.001
North China	45 (5.7)	4181 (10.6)		
Northeast China	3 (0.4)	2000 (5.1)		
East China	374 (47.8)	13136 (33.5)		
Central China	119 (15.2)	5132 (13.1)		
South China	95 (12.1)	7190 (18.3)		
Southwest China	92 (11.7)	4470 (11.4)		
Northwest China	55 (7.0)	3160 (8.0)		
Hospital (%)			Chi-square test	<0.001
Level 1 and lower	131 (16.7)	2666 (6.8)		
Level 2	177 (22.6)	9964 (25.4)		
Level 3	475 (60.7)	26623 (67.8)		
Unknown	0 (0)	16 (0)		

### Economic burden of influenza-associated outpatient visits

The mean cost per episode for influenza outpatients was US$ 155 (SD 122), consisting of US$ 70 (SD 69) for direct medical costs, US$ 26 (SD 44) for direct non-medical costs, and US$ 59 (SD 59) for indirect costs (see Table 2). The cost weighted by population structure (by age group, region, and level of hospital) of patients registered in the National ILINet was US$ 159 (SD 240), consisting of US $75 (SD 127) for direct medical costs, US $26 (SD 67) for direct non-medical costs, and US $58 (SD 86) for indirect costs. The median costs (see Additional file 6) were lower than the mean costs.

**Table 2:** Costs per episode for influenza outpatients and associated risk factors in China, 2013 (US$)^a^

Characteristic	Direct cost		Indirect costs	Total costs	Multiple linear regression
	Medical costs	Non-medical costs			
					(95 % Confidence interval)^b^
Total (*n* = 529)	70 (69)	26 (44)	59 (59)	155 (122)	
Gender	*p* = 0.141	*p* = 0.904	*p* = 0.333	*p* = 0.814	
Female (*n* = 248)	64 (52)	28 (49)	62 (65)	153 (116)	Reference
Male (*n* = 281)	75 (81)	25 (40)	57 (52)	157 (127)	−6 (−24,12)
Age group (years)	*p* = 0.062	*p* < 0.001	*p* = 0.006	*p* = 0.003	
< 5 (*n* = 122)	85 (84)	36 (53)	75 (75)	196 (152)	Reference
5–14 (*n* = 232)	63 (68)	28 (43)	61 (61)	153 (121)	−52 (−89,−22)^f^
15–59 (*n* = 160)	68 (59)	17 (39)	45 (34)	130 (91)	−81 (−119,−52)^f^
≥ 60 (*n* = 15)	61 (41)	10 (13)	58 (41)	129 (62)	−104 (−151,−63)^f^
Risk status^c^	*p*=0.006	*p*=0.228	*p*=0.163	*p*=0.009	
Low risk (*n* = 412)	64 (57)	26 (44)	57 (54)	146 (111)	Reference
High risk (*n* = 117)	90 (98)	28 (45)	68 (71)	186 (151)	40 (16,76)^f^
Area	*p*_=_ 0.197	*p*_=_ 0.406	*p* < 0.001	*p* < 0.001	
Urban area (*n* = 438)	69 (62)	25 (42)	67 (61)	161 (119)	Reference
Rural area (*n* = 91)	72 (96)	33 (56)	23 (23)	128 (132)	−26 (−53,14)
Region	*p* = 0.013	*p* = 0.403	*p* < 0.001	*p* = 0.004	
East China (*n* = 292)	72 (67)	26 (48)	71 (64)	169 (124)	Reference
North China (*n* = 17)^d^	82 (53)	13 (20)	38 (19)	133 (78)	−45 (−95,−3)^f^
Central China (*n* = 52)	69 (65)	20 (27)	40 (36)	129 (95)	−64 (−98,−31)^f^
South China (*n* = 64)	48 (45)	27 (45)	45 (37)	120 (100)	−51 (−78,−22)^f^
Southwest China (*n* = 68)	65 (48)	26 (37)	50 (67)	140 (103)	−38 (−68,−8)^f^
Northwest China (*n* = 36)	97 (132)	38 (52)	44 (46)	179 (191)	−5 (−52,71)
Hospital	*p* < 0.001	*p* = 0.745	*p* = 0.002	*p* = 0.002	
Level 3 (*n* = 298)	73 (60)	28 (50)	66 (67)	167 (124)	Reference
Level 2 (*n* = 119)	78 (100)	22 (35)	56 (45)	156 (138)	3 (−21,33)
Level 1 and lower (*n* = 112)	51 (48)	26 (38)	45 (43)	122 (88)	−34 (−61,−10)^f^
Virus type	*p* = 0.868	*p* = 0.973	*p* = 0.220	*p* = 0.565	
Untyped^e^ (*n* = 307)	71 (79)	25 (42)	60 (62)	157 (130)	Reference
Influenza A (*n* = 164)	67 (53)	27 (50)	61 (56)	156 (106)	−10 (−31,13)
Influenza B (*n* = 58)	70 (57)	27 (42)	47 (41)	144 (119)	2 (−25,41)

^a^Mean (standard deviation) was presented to facilitate their use in economic evaluations despite the skew in sample distributions of costs. However, Rank-sum test was used for comparing two samples, and Kruskal-Wallis test was used for comparing three or more groups because the cost distribution is too right skewed

^b^Compared to the reference, absolute increase or decrease of the total cost (in US$). And we obtained the bias-corrected and accelerated (BCa) bootstrap percentile confidence interval using the R function “boot.ci”

^c^Risk status: high risk patients refer to those with underlying medical conditions including: chronic respiratory disease, asthma, chronic cardiovascular diseases, diabetes, chronic liver disease, and chronic renal disease, etc. Other patients without these underlying diseases are low risk patients

^d^North China: 2 patients from Northeast China were grouped into North China

^e^Untyped: Laboratory tests for influenza virus type identification were not conducted

^f^ *p* < 0.05: significant differences

Mean cost per episode was significantly higher (*p* < 0.05) in patients with underlying medical conditions, urban patients, and patients who sought treatment in tertiary hospitals. There were also significant (*p* < 0.05) differences between age groups (with the highest cost observed in those aged below five years) and region (with higher costs observed in Northwest and East China). No significant association was observed between cost and gender or influenza virus type. In the multiple linear regression analysis, underlying diseases, age, region, and level of hospital were significantly associated with the total cost per episode (*p* < 0.05). The presence of underlying illnesses was found to increase the total cost per episode by US$ 40 (95 % confidence interval, 95 % CI 16–76). Seeking medical care in primary hospitals was found to decrease the total cost per episode by US$ 34 (95 % CI 10–61). Compared to the costs of outpatients from East China, the costs of outpatients from North, Central, South, and Southwest China were lower by US$ 38–64. Age was negatively associated with the total cost (see Table 2).

### Economic burden of influenza-associated hospitalizations

Inpatient costs were nearly nine times greater than outpatient costs, with an average cost per episode of US$ 1,511 (SD 1,465), consisting of US $1,038 (SD 1,315) for direct medical costs, US$ 277 (SD 288) for direct non-medical costs, and US$ 197 (SD 169) for indirect costs (see Table 3). The weighted cost was US$ 1,605 (SD 2,446), consisting of US$ 1,119 (SD 2,051) for direct medical costs, US$ 283 (SD 401) for direct non-medical costs, and US$ 203 (SD 308) for indirect costs.

**Table 3:** Costs per episode for influenza inpatients and associated risk factors in China, 2013 (US$)^a^

Characteristic	Direct cost		Indirect costs	Total cost	Multiple linear regression
	Medical cost	Non-medical cost			(95 % Confidence interval)^b^
Total (*n* = 254)	1038 (1315)	277 (288)	197 (169)	1511 (1465)	
Gender	*p*=0.479	*p*=0.863	*p*=0. 295	*p*=0. 707	
Female (*n* = 107)	1131 (1794)	295 (361)	201 (160)	1627 (1927)	Reference
Male (*n* = 147)	969 (806)	263 (220)	194 (176)	1427 (1004)	−215 (−688,116)
Age group (years)	*p* < 0.001	*p* = 0.046	*p* = 0. 018	*p* < 0.001	
< 5 (*n* = 144)	980 (775)	315 (336)	213 (178)	1508 (1021)	Reference
5–14 (*n* = 79)	1031 (2014)	229 (197)	157 (150)	1417 (2112)	−52 (−402,566)
15–59 (*n* = 27)	1175 (899)	219 (207)	227 (168)	1621 (1059)	88 (−529,639)
≥ 60 (*n* = 4)	2300 (1539)	230 (234)	205 (73)	2735 (1821)	88 (−529,639)^c^
Risk status^d^	*p* < 0.001	*p*=0.001	*p* < 0.001	*p* < 0.001	
Low risk (*n* = 120)	810 (661)	220 (179)	159 (132)	1189 (814)	Reference
High risk (*n* = 134)	1241 (1677)	328 (351)	231 (190)	1800 (1820)	617 (293,1290)^f^
Area	*p* = 0. 797	*p* = 0. 285	*p* < 0.001	*p* = 0.330	
Urban area (*n* = 171)	949 (760)	259 (270)	251 (177)	1486 (958)	Reference
Rural area (*n* = 83)	1221 (2022)	313 (320)	86 (70)	1620 (2169)	190 (−170,950)
Region	*p* = 0.041	*p* = 0.518	*p* = 0.020	*p* = 0.089	
East China (*n* = 82)	888 (749)	293 (362)	253 (215)	1433 (1019)	Reference
North China (*n* = 31)^e^	1256 (930)	350 (339)	212 (149)	1817 (1132)	505 (−15,1163)
Central China (*n* = 67)	983 (919)	252 (232)	162 (138)	1397 (1150)	26 (−310,456)
South China (*n* = 31)	896 (655)	251 (224)	172 (137)	1318 (833)	−135 (−576,308)
Southwest China (*n* = 24)	892 (986)	237 (197)	148 (119)	1277 (1157)	−82 (−582,513)
Northwest China (*n* = 19)	1935 (3766)	267 (193)	158 (109)	2360 (3809)	1076 (−118,4562)
Hospital	*p* = 0.022	*p* = 0.400	*p* = 0. 182	*p* = 0.040	
Level 3 (*n* = 177)	1090 (1422)	270 (240)	203 (168)	1563 (1527)	Reference
Level 2 (*n* = 58)	894 (750)	283 (401)	202 (189)	1379 (1065)	−83 (−618,261)
Level 1 and lower (*n* = 19)	989 (1633)	316 (303)	130 (93)	1436 (1925)	−213 (−1362,1101)
Virus type	*p* = 0.300	*p* = 0.029	*p* = 0.074	*p* = 0.178	
Untyped^g^ (*n* = 184)	1083 (1462)	292 (294)	212 (182)	1587 (1611)	Reference
Influenza A (*n* = 34)	953 (969)	222 (201)	171 (132)	1346 (1114)	−98 (−536,320)
Influenza B (*n* = 36)	887 (636)	250 (321)	145 (112)	1282 (817)	−481 (−996,−69)^f^

^a^Mean (standard deviation) was presented to facilitate their use in economic evaluations despite the skew in sample distributions of costs. However, Rank-sum test was used for comparing two samples, and Kruskal-Wallis test was used for comparing three or more groups because the cost distribution is too right skewed

^b^Compared to the reference, absolute increase or decrease of the total cost (in US$). And we obtained the bias-corrected and accelerated (BCa) bootstrap percentile confidence interval using the R function “boot.ci”

^c^4 patients in the ≥60 age group were grouped into 15–59 years of age group in the multivariable linear regression analysis

^d^Risk status: high risk patients refer to those with underlying medical conditions including: chronic respiratory disease, asthma, chronic cardiovascular diseases, diabetes, chronic liver disease, and chronic renal disease, etc. Other patients without these underlying diseases are low risk patients

^e^North China: 1 patients from Northeast China were grouped into North China

^f^ *p* < 0.05: significant differences

^g^Untyped: Laboratory tests for influenza virus type identification were not conducted

Direct medical costs for inpatients constituted a larger proportion of the total costs than for outpatients (69 % vs. 45 %). Inpatient costs (urban: US$ 1,486; rural: US$ 1,620) corresponded to 31 and 113 % of the annual per capita income in urban (US$ 4,769) and rural (US$ 1,436) areas, respectively. There were significant differences in costs in terms of underlying medical conditions (*p* < 0.05). Although the differences of total costs were not observed among geographic regions, the indirect costs in East China (mean US$ 253) were significantly higher than those in other regions (mean US$ 148–212) (*p* < 0.05). Multiple linear regression analysis revealed that underlying illnesses increased the total cost per episode by US$ 617 (95 % CI 293–1,290) (see Table 3).

The economic burden of influenza-associated hospitalizations in Jingzhou, Hubei province was US$ 8.64–10.04 million among children ≤ five years of age, US$ 2.15–3.41 million among the elderly, and US$ 14.99–17.31 million among the total population (n = 5.71 million) in 2013. Young children and the elderly accounted for over 70 % of this burden.

### Sensitivity analysis on the economic burden of influenza-associated outpatient visits and hospitalization

We found that 35.3 and 60.6 % of outpatient and inpatient interviewees, respectively, were unable to precisely recall at least one piece of information about costs and gave ranges instead. When the upper limits rather than the lower limits of the ranges (which were used in the baselineanalysis above) were used, the total costs rose by 12 and 8 % for outpatients and inpatients, respectively.

### Cost validation

All 38 patients in the cost validation survey were younger than 16 years of age and came from tertiary hospitals. Among them, 84.0 % were urban residents. Differences in characteristics of inpatients by gender, underlying diseases, and length of stay in hospital were not significant between the cost validation survey and telephone study (*p* > 0.05). For these patients, the average direct medical costs obtained from the telephone survey in the baseline analysis (US$ 977, SD 636) and in the sensitivity analysis (US$ 1,076, SD 828) were 18 and 30 % higher, respectively, than those obtained from the medical records (US$ 830, SD 456). When we adjusted for recall bias for costs in the baseline analysis, the costs came to be US$ 131 and US$ 1,281 for outpatients and inpatients, respectively.

## Discussion

Our retrospective telephone survey found that seasonal influenza poses a substantial economic burden for patients in mainland China, particularly for young children, the elderly, and patients with underlying medical conditions. The mean costs per influenza episode for outpatients and inpatients were US$ 155 (SD 122) and US$ 1,511 (SD 1,465), respectively. Costs associated with urban and rural inpatients corresponded to 31 and 113 % of the annual per capita income in urban and rural areas, respectively. Considering only influenza hospitalizations, we found that the economic burden of patients in Jingzhou city (which has 0.42 % of China’s population) was US$ 14.99–17.31 million in 2013. We are now conducting a survey to estimate the influenza-related outpatient incidence in China so we can get an accurate picture of the total economic burden associated with influenza at the national level.

Influenza mostly affected children aged below five years, the elderly, and those with underlying diseases. Among the outpatients, costs were highest for children aged below five years (US$ 196 vs. US$ 129–153 for other age groups). Among the inpatients, costs were highest among those aged 60 years or above (US$ 2,735 vs. US$ 1,417–1,621 for other age groups). We found that young children and the elderly accounted for over 70 % of the economic burden of influenza-associated hospitalizations in Jingzhou in 2013. In agreement with previous studies [6, 7], our study indicated that both inpatients and outpatients with underlying medical conditions had higher economic burdens than patients without underlying medical conditions. The presence of underlying illnesses was found to increase the total cost per influenza episode by US$ 40 (95 % CI 16–76) and US$ 617 (95 % CI 293–1,290) for outpatients and inpatients, respectively. This finding supports the current influenza immunization policies in China, as well as the WHO recommendations, which advise on the immunization of young children, the elderly, and those in clinical risk groups.

Our estimates of influenza-related direct inpatient costs were similar to those of a study done in Hong Kong (US$ 1,523–1,661) [21], but much lower than those reported in the US study (US$ 13,768–103,252) [22]. Our direct outpatient medical cost estimates were also lower than the figures in the US study (US$ 120–928) [22]. Differences in costs could be partially due to differences in study methodologies. For example, the US study included all cases with pneumonia and influenza diagnostic codes rather than just laboratory-confirmed influenza patients. Also, costs were extracted from a health insurance claims database for a much longer period (two weeks prior to the date of admission to 30 days post-discharge) [22]. Apart from this, the differences may be explained by actual differences in healthcare labor costs between the two countries, as well as in healthcare financing (as patients in China have to pay out-of-pocket for a larger portion of the total medical costs than in the US) [23].

The study in the US reported that indirect costs (productivity losses) were about 10 times higher than direct medical costs [22]. However, our survey found that direct medical costs were the dominant cost driver for influenza patients in China. Differences in methodology and income may in part account for this discrepancy. For example, in the US study, days of productivity loss were calculated using the average daily wage of US$ 145 [22], which is much higher than our estimates of US$ 12 and US$ 4 for urban and rural areas of China, respectively.

Prior to our study, several regional studies have been published on the economic burden associated with laboratory-confirmed influenza cases in China (by checking medical records). As mentioned above, the mean costs obtained from the telephone survey were 18 % higher than those obtained from medical records. Accordingly, we decreased our costs by 18 % when we compared the region-specific costs with previous estimates. Direct outpatient medical costs estimated in our study were similar to those reported in a previous study done in Zhuhai city, Guangdong province (mean: US$ 28.0 vs. US$ 26.0) [5], while the direct inpatient medical costs were 1.5 times greater than those reported in a study conducted in the provincial capitals of Sichuan, Hunan, and Shandong provinces [7]. Some of the differences in costs may partially stem from variations in patient characteristics. For instance, in the Sichuan, Hunan, and Shandong study, the proportion of included patients with underlying medical conditions was only 20.8 %, much lower than in our study (46.0 %) [7]. When using the same weights for the proportion of the population with underlying diseases, the direct inpatient medical costs in the previous study were similar to that in our study (US$ 858 vs. US$ 891). Only one previous study assessed influenza-related indirect costs. This study, done in Suzhou city, Jiangsu province, reported indirect costs of US$ 40.8 for illness in children aged below five years in the outpatient setting [9], which was lower than our estimation (US$ 50.8).

Our study had several limitations. First, as it was a retrospective study, recall bias was inevitable. To minimize its effect, we selected each participant’s most recent influenza episode. The time between the influenza episode and interview was less than 46 days for 50 % of our study participants. To partially test for recall bias, we conducted a sensitivity analysis and a cost validation survey. The sensitivity analysis found that the lower limits of ranges of costs reported by patients were relatively closer to the actual cost than the upper limits. Despite this, the lower limits of the cost ranges overstated the actual costs by 18 %.

Second, our samples were from the National ILINet and SARINet. There were statistically significant differences in age, region, and hospital level between influenza patients who participated in the present study and those who didn’t. To generalize our results to represent the costs of all influenza patients registered in the National ILINet and SARINet, we weighted the costs by age group, region, and level of hospital using the population structure of the surveillance networks as a reference. The weighted costs increased by 2.6 and 6.2 % for influenza outpatients and inpatients, respectively. This revealed that the differences in demographic characteristics did not have much of an impact on costs. However, due to a lack of characteristics of influenza patients at the national level, we couldn’t precisely determine whether our sample represents all influenza patients in China. Instead, we evaluated the representativeness of hospitals in the National ILINet and SARINet for all medical institutions in China. This could partially account for the representativeness of our samples. We found that the high-level hospitals were overrepresented in the National ILI and SARI sentinel hospitals, and that the two surveillance systems do not cover basic medical institutions (see Additional file 1). Patients with severe influenza are more likely to seek medical help in high-level hospitals, while patients with mild influenza prefer basic medical institutions or practice self-care at home. As such, patients with severe influenza may be overrepresented in our survey, which may result in an overestimation of the economic burden associated with influenza outpatient visits and hospitalizations. Finally, only 15 outpatients and four inpatients were over 60 years of age. Hence, the estimation of influenza-related economic burden of elderly patients may not be totally accurate.

Despite these limitations, the present study provides a more nationally representative cost estimate than previous studies, as well as a comprehensive assessment of both the direct and indirect costs of influenza outpatients and inpatients in China. Characterizing the economic burden associated with influenza is a cornerstone of cost-effectiveness studies, which are crucial for policy decision-making in influenza vaccination programs. A previous systematic review showed that 43 % of cost-effectiveness studies (22/51) reported that influenza vaccination saves costs, and the majority of the remaining studies reported that influenza vaccination was cost-effective, with cost-effectiveness ratios below $50,000/QALY (quality-adjusted life-year) [24]. These studies demonstrate that influenza vaccination is economically favorable for children and the elderly [24]. Although a comprehensive cost-effectiveness analysis of influenza vaccination is still lacking in mainland China, conclusions from studies and reviews done in other countries consistently suggest that influenza vaccination is likely to be cost-effective. The present study forms the basis to conduct cost-effectiveness analyses in the future, in order to assess the reduction in disease and economic burden associated with widespread seasonal influenza vaccination in China.

## Conclusion

Our survey found that the economic burden of influenza-associated outpatient and inpatient visits in China is substantial, particularly for young children, the elderly, and patients with underlying medical conditions. A recent systematic review found that information about costs associated with influenza and cost-effectiveness of influenza vaccination is severely lacking in low- and middle-income countries [24]. Our study is important to fill this evidence gap, as well as to provide information on the burden of disease and the cost-effectiveness of seasonal influenza vaccination in China and other middle-income countries. Such studies are vital for informing evidence-based decision-making in vaccination policies in these settings.

## Additional files

Additional file 1:
**Representativeness of the National Influenza-like Illness Surveillance System.** (DOCX 21 kb) Additional file 2:
**Telephone survey questionnaire.** (DOCX 22 kb) Additional file 3:
**The Compiling Rules of Zoning and Urban-Rural Division Code for Statistics in China.** (DOCX 18 kb) Additional file 4:
**Definition of hospital levels.** (DOCX 19 kb) Additional file 5:
**Annual income per capita in urban and rural areas by province in China.** (DOCX 19 kb) Additional file 6:
**Economic burden of influenza outpatients and inpatients (medians, interquartile ranges).** (DOCX 30 kb)

## Abbreviations

CATI: Computer-assisted telephone interviewing system
CDC: Center for Disease Control and Prevention
ILI: Influenza-like illness
ILINet: Influenza-like Illness Surveillance Network
IQR: Interquartile range
RT-PCR: Reverse transcription polymerase chain reaction
SD: Standard deviation
SARI: Severe acute respiratory infections
SARINet: Severe Acute Respiratory Infections Sentinel Surveillance Network
WHO: World Health Organization
