# Supplementary material for: The economic burden of influenza-associated outpatient visits and hospitalizations in China: a retrospective survey
Source: Infect Dis Poverty. 2015 Oct 6;4:44. doi: 10.1186/s40249-015-0077-6 (PMC4595124; doi:10.1186/s40249-015-0077-6)
Supplement: Additional file 1: — Representativeness of the National Influenza-like Illness Surveillance System. (DOCX 21 kb) [file 40249_2015_77_MOESM1_ESM.docx]

**Representativeness of National Influenza-like Illness Surveillance System**

The National Influenza-like Illness (ILI) Surveillance Network was established to monitor the activity of as well as antigenic and genetic changes in seasonal influenza in China. This network includes 408 provincial- and prefecture-level Centers for Disease Control and Prevention (CDC) and 554 sentinel hospitals distributed in 31 provinces.

1. Type of sentinel hospitals

ILI patients may seek medical care in the following medical institutions:

1. Hospital: General Hospital, Hospitals Specialized in Traditional Chinese Medicine, Hospitals of Integrated Traditional Chinese with Western Medicine, Nationalities Hospital, Specialized Hospital (e.g., Otolaryngology Hospital, Children Hospital, Hospital for Infectious Diseases, Obstetrical and Gynecological Hospital, Cardiovascular Hospital)
2. Basic Medical Institutions: Community Health Service Centers, Health Centers, Clinics, and Outpatient Department
3. Public Health Agencies: Women and Children Care Agencies

From Chinese Health Statistic Yearbook 2013, we obtained the number of each type of above medical institutions in China. We found that the distribution of type of ILI sentinel hospitals is significantly different from that of all above medical institutions in China. Of 554 ILI sentinel hospitals, 88.4% (n=490) are General Hospitals, over-representative of General Hospitals. The Nationalities Hospital and Basic Medical Institutions are not included in ILI sentinel hospitals. (Table 1s) In addition, ILI sentinel hospitals only include three types of Specialized Hospital, i.e., Children Hospital, Hospital for Infectious Diseases, and Obstetrical and Gynecological Hospital, but do not cover such Specialized Hospital as Otolaryngology Hospital and Cardiovascular Hospital.

2. Region of sentinel hospitals

In 2013, there are 22 provinces, 5 autonomous regions and 4 municipalities in mainland China. Among 22 provinces and 5 autonomous regions, there are 333 prefecture-level cities. Every prefecture-level city and municipality has at least one ILI sentinel hospital. Moreover, Xinjiang Production and Construction Corps, and other two counties directly under the Province Government also have one ILI sentinel hospital, respectively.

Of the 333 prefecture-level cities, 66.4% (n=221) separately have one ILI sentinel hospital, 21.9% (n=73) separately have two ILI sentinel hospitals, and the City of Xi’an has the most ILI sentinel hospitals (n=7). Of 4 municipalities, there are 18 ILI sentinel hospitals in Shanghai, 11 in Beijing, 9 in Tianjin and 7 in Chongqing.

We further compared the geographic regions of ILI Sentinel Hospitals to that of all medical institutions in China. No significant differences of regions are observed between them. (Table 1s)

3. Level of sentinel hospitals

The levels of ILI Sentinel Hospitals are significantly different from that of all medical institutions in China. 64.1% (n=355) of ILI Sentinel Hospitals are tertiary hospitals, and only 5.6% (n=31) are level 1 and lower hospitals. The ILI Sentinel Hospitals are over-representative of high-level hospitals and under-representative of low-level hospitals.

In conclusion, the ILI Sentinel Hospitals have good geographic representativeness, but do not cover Basic Medical Institutions, and over-representative for the high-level hospitals.

**Table 1s. The characteristics of ILI sentinel hospitals and all medical institutions in China**

|  | ILI sentinel hospitals  (n=554) | All medical institutions in China  (n=934999)* | Chi-square test | P value |
| --- | --- | --- | --- | --- |
| Hospital type |  |  |  |  |
| General Hospital | 490 (88.4%) | 15021 (1.6%) | 28063.960 | <0.001 |
| Hospitals Specialized in  Traditional Chinese Medicine | 3 (0.5%) | 2889 (0.3%) |  |  |
| Hospitals of Integrated Traditional  Chinese with Western Medicine | 4 (0.7%) | 312 (0.0%) |  |  |
| Nationalities Hospital | 0 (0.0%) | 208 (0.0%) |  |  |
| Specialized Hospital | 34 (6.1%) | 905 (0.1%) |  |  |
| Basic Medical Institutions | 0 (0.0%) | 912620 (97.6%) |  |  |
| Women and Children Care Agencies | 23 (4.2%) | 3044(0.3%) |  |  |
| Region |  |  |  |  |
| Central | 63 (11.4%) | 2705 (12.2%) | 9.300 | 0.157 |
| East | 145 (26.2%) | 5577 (25.2%) |  |  |
| North | 84 (15.2%) | 3580 (16.1%) |  |  |
| Northeast | 54 (9.7%) | 2319 (10.5%) |  |  |
| Northwest | 78 (14.1%) | 2435 (11.0%) |  |  |
| South | 51 (9.2%) | 1772 (8.0%) |  |  |
| Southwest | 79 (14.3%) | 3784 (17.1%) |  |  |
| Hospital level (in descending order) |  |  |  |  |
| Level 3 | 355 (64.1%) | 1571 (7.1%) | 2355.771 | <0.001 |
| Level 2 | 168 (30.3%) | 6826 (30.8%) |  |  |
| Level 1 and lower | 31 (5.6%) | 13773 (62.1%) |  |  |

***** The Nationalities Hospital and Basic Medical Institutions are not included in ILI sentinel hospitals. Therefore, when we compared the region and hospital level, we did not consider them.
