# Supplementary material for: The economic burden of influenza-associated outpatient visits and hospitalizations in China: a retrospective survey
Source: Infect Dis Poverty. 2015 Oct 6;4:44. doi: 10.1186/s40249-015-0077-6 (PMC4595124; doi:10.1186/s40249-015-0077-6)
Supplement: Additional file 2: — Telephone survey questionnaire. (DOCX 22 kb) [file 40249_2015_77_MOESM2_ESM.docx]

**Questionnaire (brief version)**

| **No.** | **Verifying below information (recorded in the influenza surveillance network) before interviewing** |
| --- | --- |
| V1 | Is the name of the patient xxx（the name of patients）？1) Yes 2）No |
| V2 | Are you xxx（the name of patients or contacts persons of the patients）？1) Yes 2）No |
| V3 | What’s the relationship between you and the patients?  1）parents；2）grandparents； 3）brothers/sisters；4）Patient himself/herself；5）other |
| V4 | Are you/your child (grandchild/brothers/sisters) xx years old? 1) Yes 2）No |
| V5 | Did you/your child (grandchild/brothers/sisters) suffer from influenza in xx month xx year? 1) Yes 2）No |
| V6 | Do you/ your child (grandchild/brothers/sisters) live in xxxxx (address of the patient)? 1) Yes 2）No |
| If above information is verified, the patients should be included and answer following questions. | |

| **No.** | **Questions and options** | |
| --- | --- | --- |
| 1 | Did you/your child (grandchild/brothers/sisters) (for brevity, we omit “your child (grandchild/brothers/sisters” hereafter) have below chronic illnesses diagnosed by doctors when you suffer from that episode of influenza?  1）chronic respiratory disease (COPD)； 2）asthma；3）diabetes； 4）chronic cardiac disease (e.g., congenital heart disease, ischemic heart disease)； 5）chronic renal disease (e.g., chronic renal failure)； 6）chronic liver disease (e.g., cirrhosis)； 7）chronic neurological disease (e.g., stroke with persistent neurological deficit)； 8）chronic hematological disorder； 9）immune compromise as a result of disease or treatment； 10) obesity； 11）tuberculosis； 12）others； 13）no | |
| 2 | The duration of that episode: days? | |
| 3 | Did you seek medical help in the outpatient department during that episode？1）Yes 2）No （if no，skip to Question 6） | |
| 4 | How many times do you seek medical help in the outpatient department during that episode? | |
| 5 | Here is the question example for one time treatment (if beyond 1 time, please answer question 5 for each time) | |
|  | 5.1 Name of the hospital where you seek medical care |  |
|  | 5.2 How much do you spend on medication and examination (including the out-of-pocket and non-out-of-pocket costs)？ | Yuan  If the interviewee cannot recall the exact cost, please ask him/her to select an option below:  1) <100; 2) 100-299; 3) 300-499; 4) 500-799; 5) 800-999; 6) 1000-1999; 7) 2000-2999; 8) ≥3000 |
|  | 5.3 How much do you (including accompanying persons) spend on transportation？ | Yuan  If the interviewee cannot recall the exact cost, please ask him/her to select an option below:  1) <10; 2) 10-29; 3) 30-49; 4) 50-99; 5)100-199; 6) ≥200; 7) If you drove to hospitals and cannot estimate the cost, please tell me the distance between your home and the hospital: kilometers，and the type of vehicles：a.car, b.motorbike, c.bike, d.others |
|  | 5.4 How many persons accompany you to hospitals（If zero, please skip to Question 6） | persons |
|  | 5.5 How many days did a person accompany you on average? | days |
| 6 | Were you hospitalized during that episode？1）Yes 2）No （if no，skip to Question 9） | |
| 7 | How many times were you hospitalized during that episode? | |
| 8 | Here is the question example for one time treatment (if beyond 1 time, please answer question 8 for each time) | |
|  | 8.1 Name of the hospital where you seek medical care |  |
|  | 8.2 How many days were you hospitalized | days |
|  | 8.3 How much do you spend on medication and examination (including the out-of-pocket and non-out-of-pocket costs)？ | Yuan  If the interviewee cannot recall the exact cost, please ask him/her to select an option below:  1) <1000; 2) 1000-1999; 3) 2000-2999; 4) 3000-3999; 5) 4000-4999; 6) 5000-9999; 7) 10000-19999; 8) 20000-29999; 9) ≥30000 |
|  | 8.4 How many persons accompany you at hospitals（If zero, please skip to Question 8.8） | persons |
|  | 8.5 How many days did a person accompany you on average? | days |
|  | 8.6 How much did an accompanying person spend on accommodation each day on average? | Yuan/Days  If the interviewee cannot recall the exact cost, please ask him/her to select an option below:  1) <100; 2) 100-199; 3) 200-299; 4)300-399; 5)400-499; 6) ≥500 |
|  | 8.7 How much did an accompanying person spend on meals each day on average? | Yuan/Days  If the interviewee cannot recall the exact cost, please ask him/her to select an option below:  1) <20; 2) 20-49; 3) 50-99; 4)100-199; 5)200-299; 6) ≥300 |
|  | 8.8 How much did you spend on your own meals each day on average? | Yuan/Days  If the interviewee cannot recall the exact cost, please ask him/her to select an option below:  1) <20; 2) 20-49; 3) 50-99; 4)100-199; 5)200-299; 6) ≥300 |
|  | 8.9 Did you hire a carer？If yes, how much did you spend on it? If no, please fill in zero. | Yuan  If the interviewee cannot recall the exact cost, please ask him/her to select an option below:  1) <200 ; 2)200-499 ; 3) 500-999; 4) 1000-1499 ; 5) 1500-1999; 6) ≥2000 |
|  | 8.10 How much do you (including accompanying persons) spend on transportation？ | Yuan  If the interviewee cannot recall the exact cost, please ask him/her to select an option below:  1) <100 ; 2) 100-199 ; 3) 200-299; 4) 300-499 ; 5) 500-999 ; 6) ≥1000; 7) If you drove to hospitals and cannot estimate the cost, please tell me the distance between your home and the hospital: kilometers, and the type of vehicles：a.car, b.motorbike, c.bike, d.others |
| 9 | How much did you spend on self-medications for the episode of influenza? | Yuan  If the interviewee cannot recall the exact cost, please ask him/her to select an option below:  1) <30 ; 2) 30-49; 3) 50-99; 4)100-199; 5) 200-499; 6)500-999; 7) ≥1000 |
| 10 | How much did you spend on nourishment for the episode of influenza? | Yuan  If the interviewee cannot recall the exact cost, please ask him/her to select an option below:  1) <30 ; 2) 30-49; 3) 50-99; 4)100-199; 5) 200-499; 6)500-999; 7) ≥1000 |
