# Supplementary material for: The economic burden of influenza-associated outpatient visits and hospitalizations in China: a retrospective survey
Source: Infect Dis Poverty. 2015 Oct 6;4:44. doi: 10.1186/s40249-015-0077-6 (PMC4595124; doi:10.1186/s40249-015-0077-6)
Supplement: Additional file 3: — The Compiling Rules of Zoning and Urban-Rural Division Code for Statistics in China. (DOCX 18 kb) [file 40249_2015_77_MOESM3_ESM.docx]

**The Compiling Rules of Zoning and Urban-Rural Division Code for Statistics in China**

According to *the Urban-rural Division Code for Statistics* (<http://www.stats.gov.cn/tjsj/tjbz/200610/t20061018_8666.html>; <http://www.stats.gov.cn/tjsj/tjbz/tjyqhdmhcxhfdm/>) issued by the National Bureau of Statistics of China, the urban and rural areas are determined based on the conditions of local constructions. The constructions include public facilities, living facilities and other similar facilities which have been completed or are under construction.

1. Urban areas include city areas and town areas.

1. City areas refer to the residential communities and other areas which are connected with the constructions of municipal/district government seat in the municipal districts, and municipality not divided into districts.
2. Town areas refer to the residential communities and other areas which are connected with the government seat in the county-level city and other towns. The areas which are not connected with above regions but have a population of permanent residents over than 3000 people are also defined as town area, such as the independent mining areas, development zone, research organizations, colleges and the stations of farms and forest farms.

2. Rural areas are the areas not mentioned before.
