# Supplementary material for: The economic burden of influenza-associated outpatient visits and hospitalizations in China: a retrospective survey
Source: Infect Dis Poverty. 2015 Oct 6;4:44. doi: 10.1186/s40249-015-0077-6 (PMC4595124; doi:10.1186/s40249-015-0077-6)
Supplement: Additional file 4: — Definition of hospital levels. (DOCX 19 kb) [file 40249_2015_77_MOESM4_ESM.docx]

**Definition of hospital levels**

According to *the Basic Standard of Medical Institutes* (<http://www.nhfpc.gov.cn/mohzcfgs/pgz/200804/18713.shtml>) issued by National Health and Family Planning Commission of China, the hospitals are broken down into three levels in China, defined as follows:

1. Level 3

1. Beds: 500 beds and above;
2. Departments: clinical departments at least include emergency department, internal medicine department, department of surgery, obstetrics and gynecology department, and department of pediatrics, etc.; medical technical departments at least include pharmacy department, clinical laboratory, radiology department, and operating room, etc.
3. Personnel: at least 1.03 health technicians and 0.4 nurses per bed; the director of each department should at least have the professional title of associate chief physicians; at least 2 clinical nutritionist; engineering technicians account for at least 1% of all health technicians;
4. Buildings: floor area is at 60 square meters per bed; usable area is at least 6 square meters per bed; etc.
5. Equipment: basic equipment (e.g., ventilator, electrocardiograph, and anesthesia machine); equipment for each bed (same with those mentioned in Level 1 hospitals); other specific equipment for the conducted diagnosis and treatment.

2. Level 2

1. Beds: 100-499 beds and above;
2. Departments: clinical departments at least include emergency department, internal medicine department, department of surgery, obstetrics and gynecology department, and department of pediatrics, etc.; medical technical departments at least include pharmacy department, clinical laboratory, radiology department, and operating room, etc.
3. Personnel: at least 0.88 health technicians and 0.4 nurses per bed; at least 3 doctors have the professional title of associate chief physicians; at least 1 doctor has the professional title of attending physician in each professional department ;
4. Buildings: floor area is at 45 square meters per bed; usable area is at least 5 square meters per bed; etc.
5. Equipment: basic equipment (e.g., ventilator, electrocardiograph, and anesthesia machine); equipment for each bed (add 1 bed lamp besides those mentioned in Level 1 hospitals); other specific equipment for the conducted diagnosis and treatment.

3. Level 1

1. Beds: 20-99 beds and above;
2. Departments: clinical departments at least include emergency department, internal medicine department, obstetrics and gynecology department, and department of preventive medicine; medical technical departments at least include pharmacy department, clinical laboratory, radiology department, and disinfection supply division.
3. Personnel: at least 0.7 health technicians per bed; at least 3 doctors , 5 doctors and technicians in such fields like pharmacy, laboratory, and radiology; at least 1 doctor has the professional title of attending physician in each professional department ;
4. Buildings: floor area is at 45 square meters per bed;
5. Equipment: basic equipment (e.g., ventilator, electrocardiograph, and anesthesia machine); equipment for each bed (e.g., 1 bed, 1.2 mattress, 1.2 quilts); other specific equipment for the conducted diagnosis and treatment.
