# Supplementary material for: The economic burden of influenza-associated outpatient visits and hospitalizations in China: a retrospective survey
Source: Infect Dis Poverty. 2015 Oct 6;4:44. doi: 10.1186/s40249-015-0077-6 (PMC4595124; doi:10.1186/s40249-015-0077-6)
Supplement: Additional file 5: — Annual income per capita in urban and rural areas by province in China. (DOCX 19 kb) [file 40249_2015_77_MOESM5_ESM.docx]

**Annual income per capita in urban and rural areas by province in China**

**Table 2s. Per capita annual income in urban and rural areas by province in China, 2013 (US$)** (<http://www.stats.gov.cn/tjsj/ndsj/2013/indexch.htm>)

| **Province** | **Urban areas** | **Rural areas** |
| --- | --- | --- |
| Beijing | 7307.4 | 2959.8 |
| Tianjin | 5755.0 | 2556.8 |
| Hebei | 3896.8 | 1469.1 |
| Shanxi | 3875.9 | 1154.6 |
| Inner Mongolia | 4354.4 | 1387.4 |
| Liaoning | 4504.0 | 1698.4 |
| Jilin | 3800.1 | 1552.9 |
| Heilongjiang | 3413.6 | 1555.0 |
| Shanghai | 7889.4 | 3162.7 |
| Jiangsu | 5670.3 | 2194.8 |
| Zhejiang | 6656.5 | 2599.6 |
| Anhui | 4036.1 | 1307.0 |
| Fujian | 5388.1 | 1805.2 |
| Jiangxi | 3704.1 | 1417.4 |
| Shandong | 4943.5 | 1714.1 |
| Henan | 3823.1 | 1368.0 |
| Hubei | 4064.3 | 1431.2 |
| Hunan | 3977.5 | 1351.3 |
| Guangdong | 5891.9 | 1883.5 |
| Guangxi | 4039.8 | 1096.1 |
| Hainan | 4022.2 | 1346.5 |
| Chongqing | 4333.8 | 1344.8 |
| Sichuan | 3856.6 | 1274.3 |
| Guizhou | 3456.2 | 877.1 |
| Yunnan | 3986.4 | 991.2 |
| Tibet | 3641.4 | 1061.8 |
| Shaanxi | 3891.3 | 1049.6 |
| Gansu | 3252.1 | 824.4 |
| Qinghai | 3572.1 | 1000.1 |
| Ningxia | 3836.1 | 1118.7 |
| Xinjiang | 3613.5 | 1177.7 |
