# Supplementary material for: The economic burden of influenza-associated outpatient visits and hospitalizations in China: a retrospective survey
Source: Infect Dis Poverty. 2015 Oct 6;4:44. doi: 10.1186/s40249-015-0077-6 (PMC4595124; doi:10.1186/s40249-015-0077-6)
Supplement: Additional file 6: — Economic burden of influenza outpatients and inpatients (medians, interquartile ranges). (DOCX 30 kb) [file 40249_2015_77_MOESM6_ESM.docx]

**Economic burden of influenza outpatients and inpatients (median, interquartile range)**

**Table 3s. Direct and indirect costs per influenza outpatient episode and associated risk factors in China, 2013 (US$),** **median (interquartile range) ^a^**

| Characteristic | Direct cost | |  | Indirect costs |  | Total costs |
| --- | --- | --- | --- | --- | --- | --- |
|  | Medical costs | Non-medical costs |  |  |  |  |
| Total (n=529) | 53 (24,97) | 10 (3,30) |  | 43 (22,73) |  | 127 (71,206) |
| Gender | *p*_=_0.141 | *p*_=_0.904 |  | *p*_=_0.333 |  | *p*_=_0.814 |
| Female (n=248) | 49 (22,90) | 10 (3,29) |  | 44 (22,73) |  | 126 (69,206) |
| Male (n=281) | 57 (29,97) | 10 (3,30) |  | 41 (21,73) |  | 127 (72,207) |
| Age group (years) | *p*=0.062 | *p*<0.001 |  | *p*=0.006 |  | *p*=0.003 |
| <5 (n=122) | 61 (32,113) | 17 (5,42) |  | 57 (23,89) |  | 157 (88,255) |
| 5-14 (n=232) | 48 (21,81) | 14 (3,35) |  | 44 (22,74) |  | 126 (64,214) |
| 15-59 (n=160) | 61 (28,95) | 7 (2,17) |  | 37 (20,63) |  | 112 (66,170) |
| ≥60 (n=15) | 48 (30,86) | 6 (2,10) |  | 37 (26,90) |  | 134 (74,188) |
| Risk status ^b^ | *p*_=_0.006 | *p*_=_0.228 |  | *p*_=_0.163 |  | *p*_=_0.009 |
| Low risk (n=412) | 48 (24,89) | 10 (3,29) |  | 43 (22,70) |  | 120 (68,202) |
| High risk (n=117) | 65 (36,117) | 16 (3,31) |  | 44 (22,89) |  | 144 (87,251) |
| Area | *p*_=_ 0.197 | *p*_=_ 0.406 |  | *p*<0.001 |  | *p*<0.001 |
| Urban area (n=438) | 57 (26,97) | 10 (3,29) |  | 48 (30,85) |  | 134 (76,209) |
| Rural area (n=91) | 44 (19,86) | 14 (2,33) |  | 14 (7,35) |  | 88 (49,174) |
| Region | *p*=0.013 | *p*=0.403 |  | *p*<0.001 |  | *p*=0.004 |
| East China (n=292) | 57 (30,97) | 10 (3,29) |  | 55 (30,91) |  | 137 (79,217) |
| North China (n=17) ^c^ | 84 (48,123) | 8 (2,17) |  | 40 (21,53) |  | 137 (70,186) |
| Central China (n=52) | 47 (19,100) | 11 (3,26) |  | 31 (19,47) |  | 99 (53,208) |
| South China (n=64) | 38 (19,65) | 8 (1,28) |  | 33 (18,65) |  | 90 (50,140) |
| Southwest China (n=68) | 59 (23,96) | 11 (3,31) |  | 32 (16,60) |  | 142 (68,185) |
| Northwest China (n=36) | 59 (36,120) | 20 (3,49) |  | 35 (20,54) |  | 122 (74,228) |
| Hospital | *p*<0.001 | *p*=0.745 |  | *p*=0.002 |  | *p*=0.002 |
| Level 3 (n=298) | 65 (28,101) | 10 (3,30) |  | 47 (22,78) |  | 138 (78,216) |
| Level 2 (n=119) | 48 (32,97) | 9 (3,27) |  | 43 (22,71) |  | 125 (70,198) |
| Level 1 and lower  (n=112) | 39 (16,70) | 13 (2,31) |  | 33 (15,60) |  | 102 (54,160) |
| Virus type | *p*=0.868 | *p*=0.973 |  | *p*=0.220 |  | *p*=0.565 |
| Untyped ^d^ (n=307) | 52 (24,97) | 10 (3,30) |  | 44 (22,74) |  | 126 (65,208) |
| Influenza A (n=164) | 57 (25,90) | 10 (3,30) |  | 43 (22,73) |  | 129 (75,204) |
| Influenza B (n=58) | 59 (32,94) | 12 (2,28) |  | 37 (18,65) |  | 125 (76,175) |

^a^ Rank-sum test was used for comparing two samples, and Kruskal-Wallis test was used for comparing three or more groups.

^b^ Risk status: high risk patients refer to those with underlying medical conditions including: chronic respiratory disease, asthma, chronic cardiovascular diseases, diabetes, chronic liver disease, and chronic renal disease, etc. Other patients without these underlying diseases are low risk patients.

^c^ North China: 2 patients from Northeast China were grouped into North China.

^d^ Untyped: Laboratory tests for influenza virus type identification were not conducted.

**Table 4s. Direct and indirect costs per episode for influenza inpatients and associated risk factors in China, 2013 (US$),** **median (interquartile range) ^a^**

| Characteristic | Direct cost | |  | Indirect costs |  | Total cost |
| --- | --- | --- | --- | --- | --- | --- |
|  | Medical cost | Non-medical cost |  |  |  |  |
| Total (n=254) | 772(476,1176) | 200 (112,338) |  | 148 (87,272) |  | 1154 (780,1812) |
| Gender | *p*_=_0.479 | *p*_=_0.863 |  | *p*_=_0. 295 |  | *p*_=_0. 707 |
| Female (n=107) | 694 (484,1156) | 194 (110,335) |  | 156(92,280) |  | 1124 (758,1896) |
| Male (n=147) | 812 (442,1186) | 201 (113,342) |  | 139 (81,240) |  | 1174 (808,1736) |
| Age group (years) | *p*<0.001 | *p*=0.046 |  | *p*=0. 018 |  | *p*<0.001 |
| <5 (n=144) | 809 (517,1163) | 215 (133,364) |  | 157 (95,292) |  | 1201 (901,1803) |
| 5-14 (n=79) | 541 (315,1073) | 169 (97,306) |  | 120 (69,208) |  | 864 (562,1414) |
| 15-59 (n=27) | 986 (691,1307) | 159 (87,272) |  | 174 (110,286) |  | 1347 (925,2060) |
| ≥60 (n=4) | 1856 (1574,2583) | 180 (70,340) |  | 193 (148,250) |  | 2223 (1906,3051) |
| Risk status ^b^ | *p*<0.001 | *p*_=_0.001 |  | *p*<0.001 |  | *p*<0.001 |
| Low risk (n=120) | 645 (371,1043) | 169 (94,305) |  | 120 (70,219) |  | 962 (679,1419) |
| High risk (n=134) | 876 (581,1323) | 230 (132,363) |  | 174 (103,292) |  | 1286 (1010,2146) |
| Area | *p*_=_0. 797 | *p*_=_0. 285 |  | *p*<0.001 |  | *p*_=_0.330 |
| Urban area (n=171) | 726 (484,1146) | 197 (107,316) |  | 207 (133,302) |  | 1143 (832,1848) |
| Rural area (n=83) | 855 (408,1225) | 201 (125,375) |  | 68 (37,110) |  | 1174 (623,1764) |
| Region | *p*=0.041 | *p*=0.518 |  | *p*=0.020 |  | *p*=0.089 |
| East China (n=82) | 630 (398,1020) | 190 (112,337) |  | 179 (111,351) |  | 1048 (722,1851) |
| North China (n=31) ^c^ | 1017 (714,1509) | 274 (155,400) |  | 171 (101,280) |  | 1464 (1075,2240) |
| Central China (n=67) | 888 (81,1145) | 195 (94,318) |  | 126 (83,218) |  | 1201 (798,1611) |
| South China (n=31) | 694 (444,1186) | 208 (114,272) |  | 129 (83,224) |  | 1111 (766,1750) |
| Southwest China (n=24) | 582 (276,1183) | 189 (134,281) |  | 116 (54,222) |  | 915 (556,1624) |
| Northwest China (n=19) | 855 (459,1459) | 194 (147,383) |  | 157 (76,200) |  | 1211 (970,2018) |
| Hospital | *p*=0.022 | *p*=0.400 |  | *p*=0. 182 |  | *p*=0.040 |
| Level 3 (n=177) | 812 (517,1162) | 201 (117,339) |  | 153 (87,280) |  | 1182 (858,1797) |
| Level 2 (n=58) | 692 (299,1235) | 174 (96,274) |  | 139 (79,265) |  | 1000 (531,1963) |
| Level 1 and lower (n=19) | 621 (234,823) | 230 (117,410) |  | 142 (54,167) |  | 971 (522,1288) |
| Virus type | *p*=0.300 | *p*=0.029 |  | *p*=0.074 |  | *p*=0.178 |
| Untyped ^d^ (n=184) | 807 (498,1182) | 211 (131,341) |  | 157(87,292) |  | 1180 (832,1878) |
| Influenza A (n=34) | 541 (311,1181) | 161 (89,269) |  | 141 (94,169) |  | 1012 (623,1644) |
| Influenza B (n=36) | 783 (379,1126) | 147 (82,324) |  | 103 (80,179) |  | 1143 (653,1468) |

^a^ Rank-sum test was used for comparing two samples, and Kruskal-Wallis test was used for comparing three or more groups..

^b^ Risk status: high risk patients refer to those with underlying medical conditions including: chronic respiratory disease, asthma, chronic cardiovascular diseases, diabetes, chronic liver disease, and chronic renal disease, etc. Other patients without these underlying diseases are low risk patients.

^c^ North China: 1 patients from Northeast China were grouped into North China.

^d^ Untyped: Laboratory tests for influenza virus type identification were not conducted.
